# Treatment outcome of patients with recurrent glioblastoma multiforme: a retrospective multicenter analysis

**DOI:** 10.1007/s11060-017-2564-z

**Published:** 2017-07-20

**Authors:** Myra E. van Linde, Cyrillo G. Brahm, Philip C. de Witt Hamer, Jaap C. Reijneveld, Anna M. E. Bruynzeel, W. Peter Vandertop, Peter M. van de Ven, Michiel Wagemakers, Hiske L. van der Weide, Roelien H. Enting, Annemiek M. E. Walenkamp, Henk M. W. Verheul

**Affiliations:** 1 Department of Medical Oncology, Cancer Center Amsterdam, VU University Medical Center, P.O. Box 7057, 1007 MB Amsterdam, The Netherlands; 2 Department of Medical Oncology, University Medical Center Groningen, Groningen, The Netherlands; 3 Department of Neurosurgery, Cancer Center Amsterdam, VU University Medical Center, Amsterdam, The Netherlands; 4 Department of Neurology, Cancer Center Amsterdam, VU University Medical Center, Amsterdam, The Netherlands; 5 Department of Radiotherapy, Cancer Center Amsterdam, VU University Medical Center, Amsterdam, The Netherlands; 6 Department of Epidemiology and Statistics, VU University Medical Center, Amsterdam, The Netherlands; 7 Department of Neurosurgery, University Medical Center Groningen, Groningen, The Netherlands; 8 Department of Radiotherapy, University Medical Center Groningen, Groningen, The Netherlands; 9 Department of Neurology, University Medical Center Groningen, Groningen, The Netherlands

**Keywords:** Recurrent glioblastoma multiforme, Treatment strategies, Treatment effectiveness, Survival outcome

## Abstract

Glioblastoma multiforme (GBM) universally recurs with dismal prognosis. We evaluated the efficacy of standard treatment strategies for patients with recurrent GBM (rGBM). From two centers in the Netherlands, 299 patients with rGBM after first-line treatment, diagnosed between 2005 and 2014, were retrospectively evaluated. Four different treatment strategies were defined: systemic treatment (SYST), re-irradiation (RT), re-resection followed by adjuvant treatment (SURG) and best supportive care (BSC). Median OS for all patients was 6.5 months, and median PFS (excluding patients receiving BSC) was 5.5 months. Older age, multifocal lesions and steroid use were significantly associated with a shorter survival. After correction for confounders, patients receiving SYST (34.8%) and SURG (18.7%) had a significantly longer survival than patients receiving BSC (39.5%), 7.3 and 11.0 versus 3.1 months, respectively [HR 0.46 (*p* < 0.001) and 0.36 (*p* < 0.001)]. Median survival for patients receiving RT (7.0%) was 9.2 months, but this was not significantly different from patients receiving BSC (*p* = 0.068*)*. Patients receiving SURG compared to SYST had a longer PFS (9.0 vs. 4.3 months, respectively; *p* < 0.001), but no difference in OS was observed. After adjustments for confounders, patients with rGBM selected for treatment with SURG or SYST do survive significantly longer than patients who are selected for BSC based on clinical parameters. The value of reoperation versus systemic treatment strategies needs further investigation.

**Electronic supplementary material:**

The online version of this article (doi:10.1007/s11060-017-2564-z) contains supplementary material, which is available to authorized users.

## Introduction

Glioblastoma multiforme (GBM) is the most common and aggressive primary brain tumor in adults. Standard treatment for patients with newly diagnosed GBM consists of maximal surgical resection followed by postoperative radiation with concomitant and adjuvant temozolomide therapy [1]. Despite this treatment, recurrence is almost inevitable and the prognosis remains poor with a median survival of 12–15 months [2]. At the time of recurrence, treatment options are limited with modest activity. Therefore, no universally held standard of care is available for recurrent GBM (rGBM).

Systemic treatment is commonly suggested for recurrence, of which nitrosoureas (*e.g*. lomustine) are mostly used. However, effectiveness of nitrosoureas-based therapy is limited, considering a progression-free survival rate at 6 months of 19% and an objective response rate of less than 10% [3]. Unfortunately, bevacizumab fails to improve overall survival in both newly diagnosed and rGBM setting [4–9]. A temozolomide rechallenge has been studied in multiple clinical trials in various schedules with mixed results [10–12]. However, recent results suggest that patients with a O^6^-methylguanine DNA methyltransferase (MGMT) promoter-methylated recurrent tumor may benefit from a temozolomide rechallenge [13].

Some patients with rGBM undergo re-irradiation, which may result in local disease control in a proportion of patients [14–18]. However, this approach is not always feasible due to the hazards of cumulative neurotoxicity.

At the time of recurrence, only a small number of patients with well-localized tumors are eligible for re-resection. While the benefits of resecting a newly diagnosed glioblastoma have been demonstrated in several studies, the benefits of a re-resection remain unclear [19, 20]. Although there are no comparative, randomized studies available, recent reports suggest the potential benefit of a re-resection with an acceptable complication rate [21–23].

Since none of the treatments for recurrence is more beneficial than the other, treatment is based on center-specific preferences and patients’ individual characteristics, such as age, performance status, tumor location, time to recurrence, and corticosteroid use [24, 25]. The aim of this retrospective analysis was to evaluate currently applied treatment strategies for patients with rGBM to get more insight in their potential benefit and the optimal approach.

## Materials and methods

### Patient selection and data collection

This study was approved by the institutional review board of the VU University Medical Center Amsterdam (VUMC). Permission for the use of anonymized patient data was given by the institutional research departments of the VUMC and University Medical Center Groningen (UMCG).

We retrospectively collected clinical data of patients treated at VUMC and UMCG, two specialized medical centers for brain tumors in The Netherlands. Patients aged 18 years and older with rGBM after first-line treatment from January 2005 to December 2014 were enrolled. To explore the benefit of a re-resection at the time of recurrence, only patients with a resection at initial presentation were included. Furthermore, patients should have at least completed chemoradiation after first-line resection. Exclusion criteria were patients with low-grade or anaplastic gliomas, secondary GBM, cerebral metastases or other brain lesions. Patient records with insufficient data documented or an inadequate follow-up were also excluded.

**Fig. 1:**
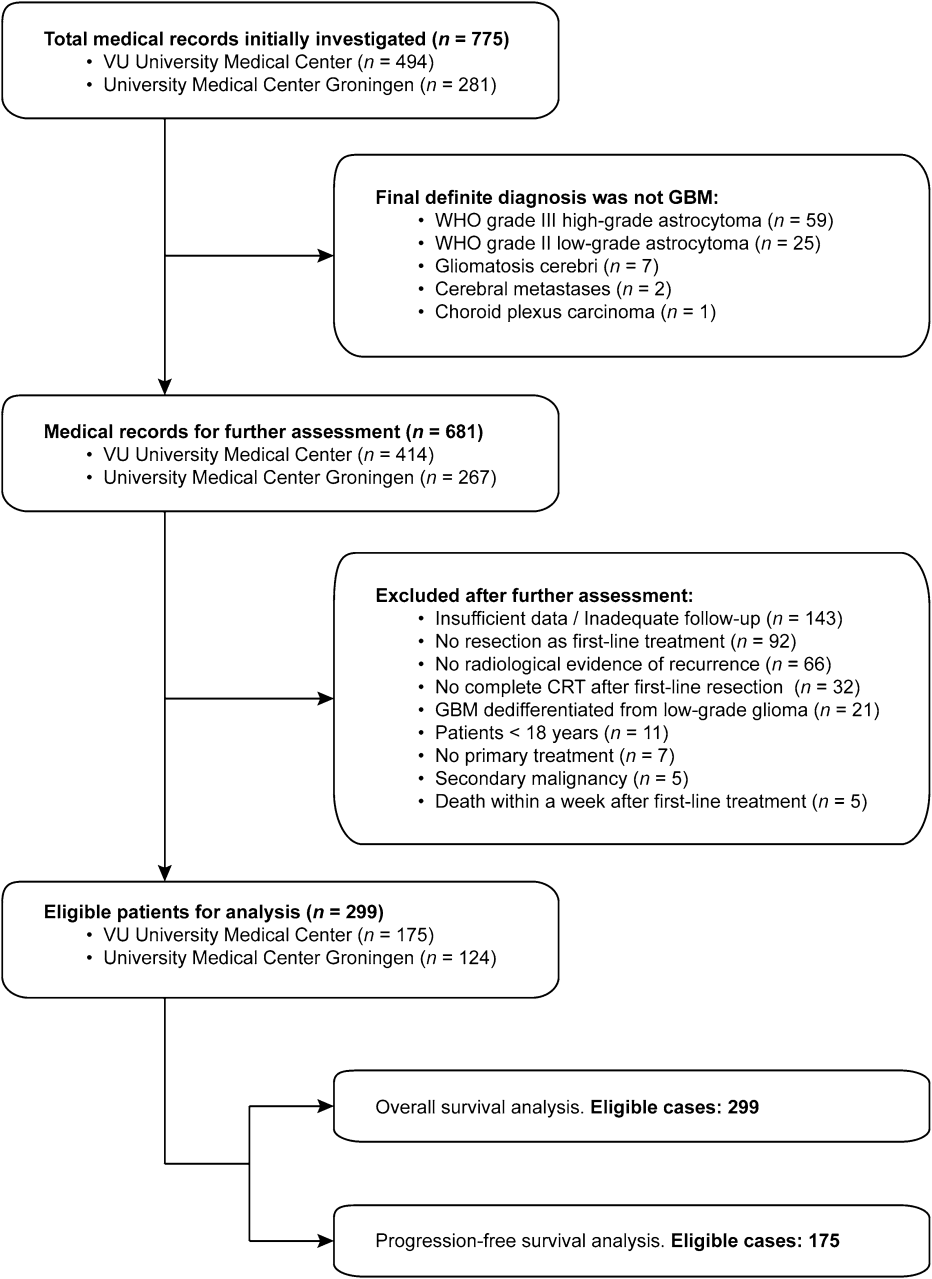
Flow chart

Eligible patients were grouped according to their treatment: systemic treatment (SYST), surgical re-resection followed by systemic treatment and/or re-irradiation (SURG), re-irradiation (RT) and best supportive care (BSC). The identification and inclusion process is illustrated in Fig. 1.

### Outcomes

Overall survival (OS) was defined as the time of objective tumor recurrence to death or considered censored at the end of follow-up. Progression-free survival (PFS) was defined as the time of objective tumor recurrence to clinical or radiological evidence of progression, death or considered censored at the end of follow-up.

### Clinical variables

Choice of treatment was included in our analyses to explore its association with survival. Age, sex, tumor extent, the extent of initial resection of contrast-enhanced lesions, Karnofsky and ECOG performance scores, corticosteroid use and time to recurrence, defined as the operation date at the time of diagnosis to the date of objective tumor recurrence, were also included in these analyses to investigate their association with survival and correct for their potential confounding effect. Furthermore, the possible interacting effect between these clinical variables and choice of therapy were investigated in our analyses. Treatment centers were considered as strata in the analyses.

### Statistical analysis

Differences between treatment groups were evaluated using an overall Chi square test for categorical data, Kruskal–Wallis ANOVA for ordinal and continuous data, and the log-rank test for censored time-to-event data. Post hoc tests with Bonferroni correction were performed to evaluate pairwise differences between treatment groups in case of a significant overall effect between treatment groups. The Kaplan–Meier method was used to calculate the median OS and PFS and to produce survival curves. The Cox proportional hazards model was used as an univariate analysis to determine significant differences between treatment groups, followed by a multivariate Cox regression analysis to adjust for confounders and possible interacting effects. Furthermore, hazard ratios (HRs) for the treatment groups and clinical variables were calculated and reported with 95% confidence intervals. Treatment centers were considered as strata in the Cox regression analysis. For all statistical analyses IBM SPSS Statistics, version 22, was used.

## Results

### Patients

A total of 681 patients with GBM were initially identified in both centers. Subsequent to reviewing the records, 299 eligible patients were included in our analyses. Main reasons for exclusion were insufficiently documented data (21.0%), no resection at initial presentation (13.5%) or no documented recurrence (9.7%).

Patient characteristics are summarized in Table 1. All patients completed chemoradiation during first-line treatment. Treatment groups differed significantly in regard to patients’ age with a median age of 62 years for the patients in the BSC group, compared to 56 years in the SURG group and 59 years in both the SYST and RT groups (overall *p* = 0.041). However, post hoc tests with Bonferroni correction did not reveal specific pairs of treatment groups that differed significantly. The percentage of men was not significantly different between treatment groups (*p* = 0.106). The rate of complete initial resections of contrast-enhanced lesions was significantly higher in the SURG group compared to the BSC group (post hoc *p* < 0.001). The time to recurrence significantly differed between treatment groups (overall *p* < 0.001). Post hoc test showed a significantly shorter time to recurrence for patients in the BSC group, a median of 263 days, compared to 376 days in the SYST group, 474 days in the SURG group and 554 days in the RT group (post hoc *p* < 0.001). ECOG and KPS performance status significantly differed between treatment groups (overall *p* < 0.001). Post hoc analyses showed worse performance scores in the BSC groups compared to all other treatment groups (post hoc *p* < 0.001*)*. The performance scores between the SYST, SURG and RT groups did not significantly differ. In addition, corticosteroids were more frequently used in the BSC group in comparison to the other groups (post hoc *p* ≤ 0.001). Furthermore, patients in the SURG group used corticosteroids less frequently compared to patients in the SYST group (post hoc *p* = 0.003). The median corticosteroid dose in the SYST group, 4 mg/day, was significantly lower than the median dose in the BSC group (6 mg/day; post hoc *p* = 0.003). Lastly, anti-epileptic drugs were used at similar rates in all treatment groups (overall *p* = 0.445).

**Table 1:** Demographic and clinical characteristics of all patients

Factor	Total study population (*n* = 299)				
	Systemic treatment (*n* = 104)	Surgical reintervention (*n* = 56)	Re-irradiation (*n* = 21)	Best supportive care (*n* = 118)	*P* value
Age (years)					0.041
Mean	56	55	57	60	
Median	59	56	59	62	
Range	19–77	26–74	26–71	21–85	
Age (no)					0.511
≤ 50	26 (25.0%)	18 (32.1%)	5 (23.8%)	23 (19.5%)	
51–65	55 (52.9%)	27 (48.2%)	10 (47.6%)	59 (50.0%)	
> 65	23 (22.1%)	11 (19.6%)	6 (28.6%)	36 (30.5%)	
Gender (no)					0.106
Male	71 (68.3%)	43 (76.8%)	10 (47.6%)	78 (66.1%)	
Female	33 (31.7%)	13 (23.2%)	11 (52.4%)	40 (33.9%)	
Tumor location (no)					0.182
Frontal lobe	28 (26.9%)	21 (37.5%)	6 (28.6%)	39 (33.1%)	
Parietal lobe	17 (16.3%)	5 (8.9%)	4 (19.0%)	12 (10.2%)	
Occipital lobe	6 (5.8%)	0 (0.0%)	0 (0.0%)	3 (2.5%)	
Temporal lobe	22 (21.2%)	16 (28.6%)	7 (33.3%)	25 (21.2%)	
Multiple lobes	30 (28.8%)	13 (23.2%)	2 (9.5%)	34 (28.8%)	
Other	1 (1.0%)	1 (1.8%)	2 (9.5%)	5 (4.2%)	
Tumor extent (no)					0.257
Single lobe	74 (71.2%)	43 (76.8%)	19 (90.5%)	84 (71.2%)	
Multiple lobes	30 (28.8%)	13 (23.2%)	2 (9.5%)	34 (28.8%)	
Extent of initial resection (no)					0.004
Complete	27 (26.0%)	23 (41.1%)	4 (19.0%)	20 (16.9%)	
Incomplete	67 (64.4%)	26 (46.4%)	15 (71.4%)	93 (78.8%)	
Unknown	10 (9.6%)	7 (12.5%)	2 (9.5%)	5 (4.2%)	
Adjuvant Temozolomide cycles (no)					<0.001
Mean	6	5	5	4	
Median	6	6	6	5	
Range	1–6	0–12	0–6	0–12	
Karnofsky performance status (no)					<0.001
100–90	42 (40.4%)	25 (44.6%)	7 (33.3%)	4 (3.4%)	
80–70	49 (47.1%)	29 (51.8%)	10 (47.6%)	37 (31.4%)	
60–50	11 (10.6%)	2 (3.6%)	4 (19.0%)	39 (33.1%)	
40–30	2 (1.9%)	0 (0.0%)	0 (0.0%)	38 (32.2%)	
ECOG performance score (no)					<0.001
0	39 (37.5%)	20 (35.7%)	6 (28.6%)	3 (2.5%)	
1	53 (51.0%)	30 (53.6%)	11 (52.4%)	35 (29.7%)	
2	9 (8.7%)	6 (10.7%)	4 (19.0%)	37 (31.4%)	
3	3 (2.9%)	0 (0.0%)	0 (0.0%)	43 (36.4%)	
Time to recurrence (days)					<0.001
Mean	459	595	598	323	
Median	376	474	554	263	
Range	113–2097	71–1540	259–1733	59–1453	
Use of steroids (no)					<0.001
Yes	61 (58.7%)	18 (32.1%)	11 (52.4%)	94 (79.7%)	
No	42 (40.4%)	35 (62.5%)	10 (47.6%)	17 (14.4%)	
Unknown	1 (1.0%)	3 (5.4%)	0 (0.0%)	7 (5.9%)	
Daily steroid dose (mg)					0.018
Mean	5	6	5	6	
Median	4	6	3	6	
Range	1–12	0–12	1–16	1–20	
Use of antiepileptic drugs (no)					0.445
Yes	58 (55.8%)	34 (60.7%)	11 (52.4%)	73 (61.9%)	
No	46 (44.2%)	20 (35.7%)	9 (42.9%)	43 (36.4%)	
Unknown	0 (0.0%)	2 (3.6%)	1 (4.8%)	2 (1.7%)	
Extent of second resection (no)					
Complete	–	16 (28.6%)	–	–	
Incomplete	–	40 (71.4%)	–	–	
Systemic treatment at recurrence (no)					0.032
Lomustine	42 (40.4%)	18 (39.1%)	–	–	
Lomustine + bevacizumab	13 (12.5%)	0 (0.0%)	–	–	
Temozolomide	14(13.5%)	13 (28.3%)	–	–	
Bevacizumab	9 (8.7%)	1 (2.2%)	–	–	
PCV	11 (10.6%)	5 (10.9%)	–	–	
Other	15 (14.4%)	9 (19.6%)	–	–	

### Treatment outcomes for all patients

**Fig. 2:**
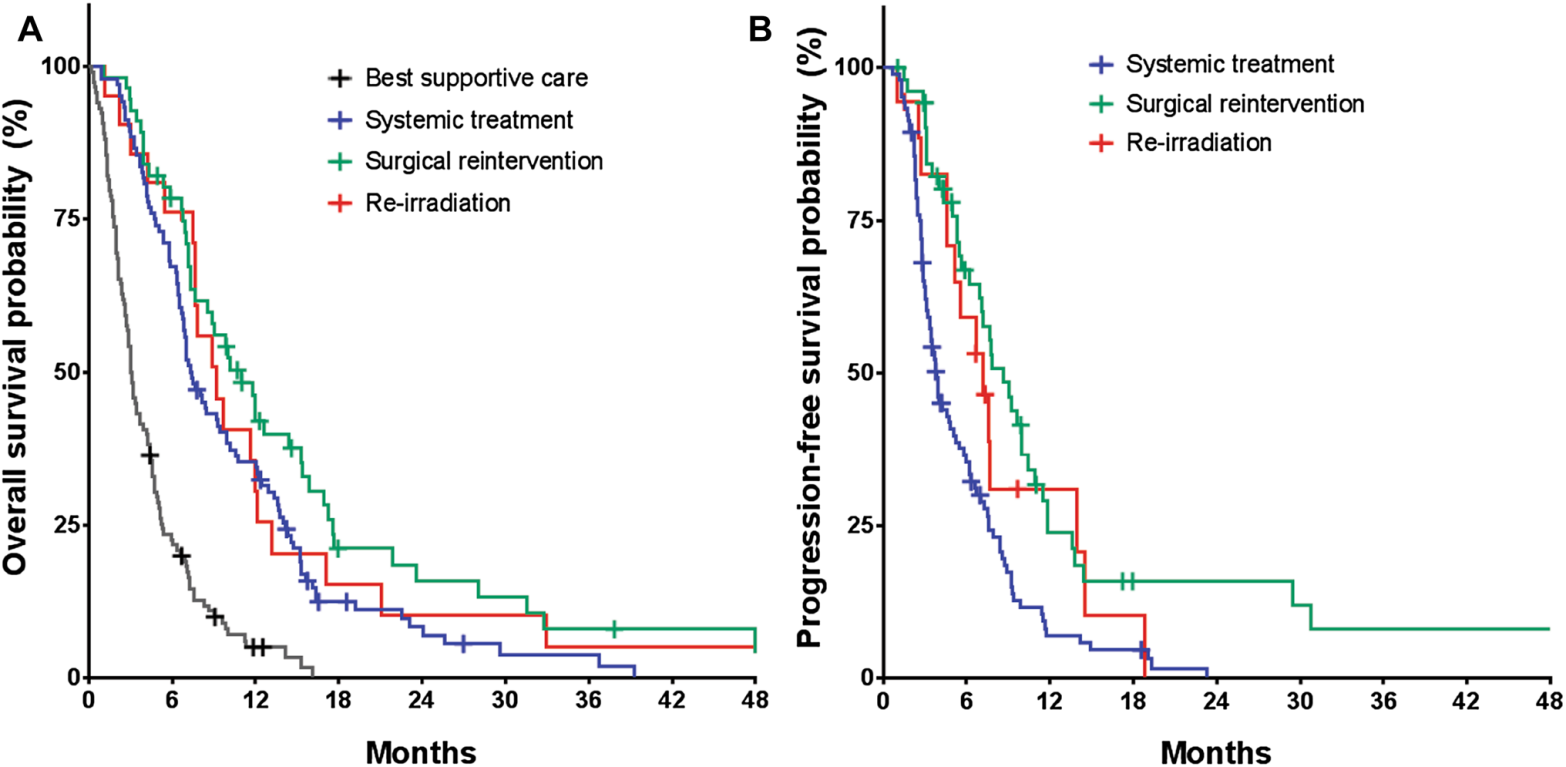
Kaplan–Meier curves of **a** overall survival and **b** progression-free survival for all patients

In Fig. 2 the survival curves for OS (A) and PFS (B) for the different treatment groups are depicted. Since follow-up data (e.g. radiological evaluation) for patients with best supportive care were unavailable, they were excluded from our PFS analysis. PFS and OS were significantly different for the treatment groups after stratification for both treatment centers (log-rank test; *p* < 0.001).

At the end of our follow-up, 7 patients (6.7%) in SYST group, 10 patients (17.9%) in SURG group, 2 patients (9.5%) in RT group and 3 patients (2.5%) in BSC group were still alive. The median OS for patients in the BSC group was 3.1 months (95% CI 2.6–3.5 months) compared to 7.3 months (95% CI 6.0–8.5 months) in the SYST group, 11.0 months (95% CI 8.2–13.8 months) in the SURG group and 9.2 months (95% CI 6.6–11.8 months) in the RT group.

In Table 2 the hazard ratios (HRs) of death are described for our analysis of OS using an univariate and multivariate Cox proportional hazards model with stratification for treatment center. In our univariate analysis overall survival did significantly differ between treatment arms (overall *p* < 0.001), with a longer OS in SYST, SURG and RT compared to the BSC group (post hoc *p* < 0.001 for all groups). Furthermore, univariate analyses of age, sex, tumor extent, extent of initial resection, performance status, time to recurrence and steroid use all showed significant associations with overall survival (*p* = 0.029, *p* = 0.001, *p* = 0.002, *p* = 0.017, *p* < 0.001, *p* < 0.001 and *p* < 0.001, respectively). Therefore, these clinical variables were included as covariates in our multivariate Cox model to correct for a possible confounding effect. Univariate analysis of tumor location (e.g. frontal, parietal, temporal or occipital lesions) showed no significant correlation with survival and was therefore excluded from our multivariate analyses (*p* = 0.100). After adjustment for the confounders, patients in the SYST group and SURG group still had a significantly prolonged survival compared to the BSC group with adjusted hazard ratios of 0.46 (95% CI 0.33–0.66; *p* < 0.001) and 0.36 (95% CI 0.23–0.58; *p* < 0.001), respectively. The survival benefit of the re-irradiation group over BSC was not significantly different after adjustment for confounders (HR = 0.60; 95% CI 0.34–1.04; *p* = 0.068). In addition to these findings, there was no significant difference in survival between the SURG and SYST group (*p* = 0.241). None of our clinical variables had an interacting effect with choice of treatment on survival.

**Table 2:** Cox proportional hazards model of overall survival after adjustments for confounders

Factor	Study population (*n* = 299)		
	No. of events/no. of patients	HR (95% CI)	*P* value
Univariate			
Treatment groups			<0.001
Best supportive care	113/118	1	
Systemic treatment	97/104	0.31 (0.23–0.42)	
Surgical reintervention	46/56	0.20 (0.13–0.29)	
Re-irradiation	19/21	0.28 (0.17–0.46)	
Multivariate analysis			
Treatment groups			<0.001
Best supportive care	113/118	1	
Systemic treatment	97/104	0.46 (0.33–0.66)	
Surgical reintervention	46/56	0.36 (0.23–0.58)	
Re-irradiation	19/21	0.60 (0.34–1.04)	
Age (years)	–	1.01 (1.00–1.02)	0.019
Sex			0.002
Male	192/202	1	
Female	83/97	0.64 (0.49–0.85)	
Tumor extent			0.002
Single lobe	201/220	1	
Multiple lobes	74/79	1.57 (1.18–2.10)	
Extent of initial resection			0.878
Incomplete	185/201	1	
Complete	67/74	0.98 (0.73–1.32)	
Recurrence-free interval (days)	–	0.999 (0.999–1.00)	0.048
ECOG performance score			0.124
0	57/68	1	
1	119/129	1.24 (0.86–1.81)	
2	55/56	1.61 (1.01–2.58)	
3	44/46	1.83 (1.06–3.16)	
Use of steroids			0.001
No	87/104	1	
Yes	179/184	1.85 (1.33–2.59)	

Based on 175 patients from the SYST, SURG and RT groups included in our analysis of PFS, 1 patient (1.0%) in the SYST group, 6 patients (10.7%) in the SURG group and 1 patient (4.8%) in the RT group had no evidence of progression at the end of our follow-up. For 12 of the 167 patients that showed progression (7.2%), the date of progression was defined as date of death. Median PFS for patients in the SYST group was 4.3 months (95% CI 3.0–5.6 months) compared to 9.0 months (95% CI 6.8–11.3 months) in the SURG group and 7.7 months (95% CI 1.8–13.5 months) in the RT group. A significant difference in progression-free survival was seen between treatment groups (overall *p* < 0.001; see Supplementary Table S1). Patients in the SURG group had a prolonged progression-free survival compared to patients in the SYST group, but not compared to patients receiving re-irradiation (post hoc *p* < 0.001 and *p* = 0.176, respectively). The difference compared to SYST remained significant after correction for confounders with an adjusted HR of 0.37 (95% CI 0.23–0.59; *p* < 0.001). However, PFS did not differ between the SYST and RT groups (*p* = 0.390).

### Center-specific outcomes

Table 3 summarizes patient data according to corresponding treatment groups and the median survival outcomes per treatment center. In VUMC significantly more patients received a re-resection prior to adjuvant treatment (25.7 vs. 8.9%; *p* < 0.001) and in UMCG significantly more patients underwent re-irradiation (14.5 vs. 1.7%; *p* < 0.001). Median OS were similar for both centers, 5.8 months (95% CI 4.6–7.0 months) versus 7.1 months (95% CI 6.5–7.7 months) in VUMC and UMCG, respectively (*p* = 0.398; see Supplementary figure S1). The median PFS in VUMC was 5.5 months (95% CI 3.9–7.0) compared to 6.2 months (95% CI 4.5–7.9) in UMCG.

**Table 3:** Center-specific outcomes

Factor	Total study population (*n* = 299)		
	VU Medical Center	University Medical Center Groningen	Both centers
Proportion of patients (no)	175/299 (58.5%)	124/299 (41.5%)	299/299 (100%)
Treatment groups (no)			
Best supportive care	66 (37.7%)	52 (41.9%)	118 (39.5%)
Systemic treatment	61 (34.9%)	43 (34.7%)	104 (34.8%)
Surgical reintervention	45 (25.7%)	11 (8.9%)	56 (18.7%)
Re-irradiation	3 (1.7%)	18 (14.5%)	21 (7.0%)
Median OS of all patients (95% CI)	5.8 months [4.6–7.0 months]	7.1 months [6.5–7.7 months]	6.5 months [5.7–7.4 months]
Median OS, excluding Best supportive care (95% CI)	7.7 months [6.0–9.3 months]	10.0 [6.8–13.2 months]	8.5 months [6.9–10.1 months]
Median PFS, excluding Best supportive care (95% CI)	5.5 months [3.9–7.0 months]	6.2 months [4.5–7.9 months]	5.5 months [4.4–6.5 months]

### Subgroup analysis: patients completing post-operative chemoradiation and adjuvant temozolomide

Subgroup analyses were performed in patients that completed chemoradiation and at least six adjuvant cycles of temozolomide after maximal surgical resection (i.e. the Stupp regimen). Survival outcomes were almost comparable for this homogenous subgroup of 199 patients (see Supplementary figure S2A and 2B; Supplementary tables S2 and S3). However, an important finding was that completed Stupp patients in the RT group had a prolonged survival compared to the BSC group, even after adjustment for confounders (*p* = 0.005). No statistical difference was seen between the SYST, SURG and RT treatment groups.

## Discussion

In this retrospective analysis of currently applied, second-line therapies, we provide a better insight in the clinical outcome of patients with rGBM. OS between the different treatment strategies (i.e. systemic treatment, re-irradiation or a surgical re-intervention followed by adjuvant therapy) did not differ significantly. As expected from other analyses, older age, tumor extent to multiple lobes and steroid use were significantly associated with a shorter survival. In addition, patients with rGBM undergoing SYST and SURG had a significantly longer survival compared to patients receiving BSC. The survival benefit of the RT group over BSC was not significantly different from patients receiving BSC, which may be due to the small size of the group. However, a subgroup analysis of patients completing the Stupp regimen did show a favorable survival outcome for RT patients compared to the BSC group. No survival benefit of SURG compared to SYST was detected, but patients receiving SURG compared to SYST did have a prolonged PFS. These findings are in line with the results of previous studies in which treatment outcomes were compared to best supportive care [25, 26].

Our results raise some important and new questions for further research. First of all, whether or not to perform a re-resection prior to adjuvant therapy, such as systemic treatment or re-irradiation, needs further prospective evaluation to better determine the actual benefits. In several other retrospective studies, either a prolonged survival of patients with rGBM following a second surgery [21–23, 27, 28], or no survival benefit after a re-resection for recurrence were reported [25, 29, 30]. In our analysis, a prolonged PFS was seen for patients in the SURG group compared to patients in the SYST group (9.0 vs. 4.3 months). Nonetheless, no statistical difference was seen in the OS of patients undergoing a re-resection prior to adjuvant treatment compared to patients with systemic treatment alone. Therefore, it is of high interest to accurately determine in a prospective trial to what extent survival can be increased by a re-resection and how it would affect the quality of life (QoL) of patients with rGBM. Until significantly better treatment options become available, QoL data are of crucial importance in shared decision making for patients with such a detrimental prognosis.

At present, there is no standard treatment strategy for patients with rGBM. Due to this lack of an universally held standard, there are national and international differences between the treatment approaches of various treatment centers. In our analyses, we compared the outcomes of the different treatment strategies used in two university medical centers, both specialized in the multidisciplinary treatment of brain tumors. Despite some expert-based preferences in treatment strategies (SURG vs. RT), our analysis does not reveal a significant difference in survival of patients from either center.

This is one of the few, recent retrospective analyses in which a multivariate strategy has been used to determine the treatment outcomes of different treatment modalities for a large group of patients with recurrent glioblastoma. However, the few retrospective analyses available did not compare the outcomes of the different salvage therapies to best supportive care [31, 32]. Furthermore, second-line systemic treatment in these studies consisted mostly or solely of a rechallenge with temozolomide, while the majority of the patients in our analysis (48.7%) received nitrosoureal derivatives, the most commonly used second-line chemotherapeutical for rGBM.

An important limitation in the analysis of treatment outcomes of patients with rGBM is that patient’ characteristics tend to be not evenly distributed among treatment groups. This is inevitable and mainly due to the fact that variables, such as age, performance status and tumor extent, influence therapeutic decision making in clinical practice. Therefore, patients undergoing surgical resection tend to have better performance status and use corticosteroids less frequently. In our multivariate analysis, we corrected for the confounding effect of these clinical relevant variables as much as possible. However, some degree of selection bias is inevitable, and therefore our results should be interpreted with some caution. Another limitation is that data on molecular characteristics, such as the MGMT methylation status, were not evaluated. Unfortunately, the MGMT promoter status in this group of patients is not tested on a routine basis in the Netherlands, because of its minimal relevance in clinical decision making. The MGMT methylation status may predict the response to alkylating agents, and may have more prognostic value than performance status or other tumor characteristics [13, 33, 34].

In prior reports, patients with different grades of gliomas, such as anaplastic astrocytoma (WHO grade III) or glioblastomas dedifferentiated from a low-grade glioma (secondary glioblastoma) were included. Due to higher prevalence of favorable mutations, such as the isocitrate dehydrogenase 1/2 (IDH1/2) gene mutations, the prognosis and survival for these patients are slightly better than for patients with de novo (primary) glioblastoma [35–37]. To prevent influence of heterogeneity on the analysis, we excluded all other types of glioma. This could explain the slightly different survival data as compared to other studies investigating all rGBM diagnoses [29, 38].

In conclusion, we here performed a retrospective, multivariate analysis evaluating treatment outcomes of patients with rGBM treated in two referral university medical centers for brain tumors over a period of nearly 10 years. After adjustments for the confounders of older age, multifocal lesions and steroid use, patients with rGBM selected for treatment with SURG or SYST do survive significantly longer than patients who are selected for BSC based on clinical parameters. The additional value of specific treatments such as re-resection, systemic treatment or re-irradiation alone remains unclear. The true value of reoperation versus systemic treatment and how it affects QoL needs further investigation in a prospective, randomized trial to what extent a re-resection can increase survival. In addition, the modest benefit from second-line treatment for patients with rGBM clearly provokes the urgent need for innovative treatment strategies that have significant impact on their QoL and survival.

## Electronic supplementary material

Below is the link to the electronic supplementary material.

Supplementary material 1 (PDF 108 KB)

Supplementary material 2 (PDF 160 KB)

Supplementary material 3 (DOCX 29 KB)
