# Supplementary figures and images for: Treatment outcome of patients with recurrent glioblastoma multiforme: a retrospective multicenter analysis
Source: J Neurooncol. 2017 Jul 20;135(1):183–92. doi: 10.1007/s11060-017-2564-z (PMC5658463; doi:10.1007/s11060-017-2564-z)

**Supplementary Fig. S1.** Kaplan-Meier curves of overall survival for all patients per center

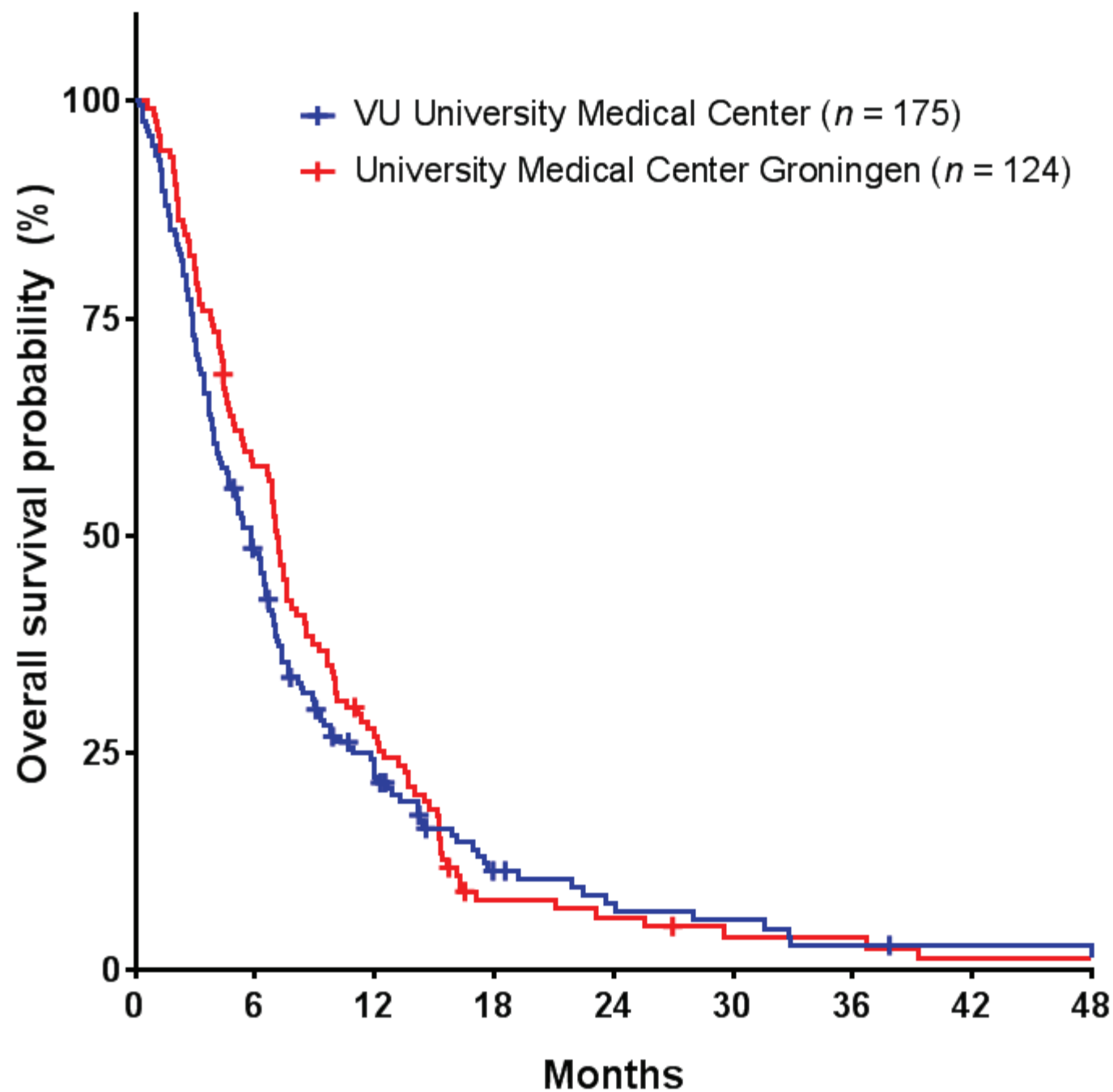

Supplement: Supplementary file 1 — Supplementary material 1 (PDF 108 KB) [file 11060_2017_2564_MOESM1_ESM.pdf]
