# Supplementary material for: Treatment outcome of patients with recurrent glioblastoma multiforme: a retrospective multicenter analysis
Source: J Neurooncol. 2017 Jul 20;135(1):183–92. doi: 10.1007/s11060-017-2564-z (PMC5658463; doi:10.1007/s11060-017-2564-z)

Supplementary Fig. S2. Kaplan-Meier curves of (A) overall survival and (B) progression-free survival for patients completing the Stupp regimen.

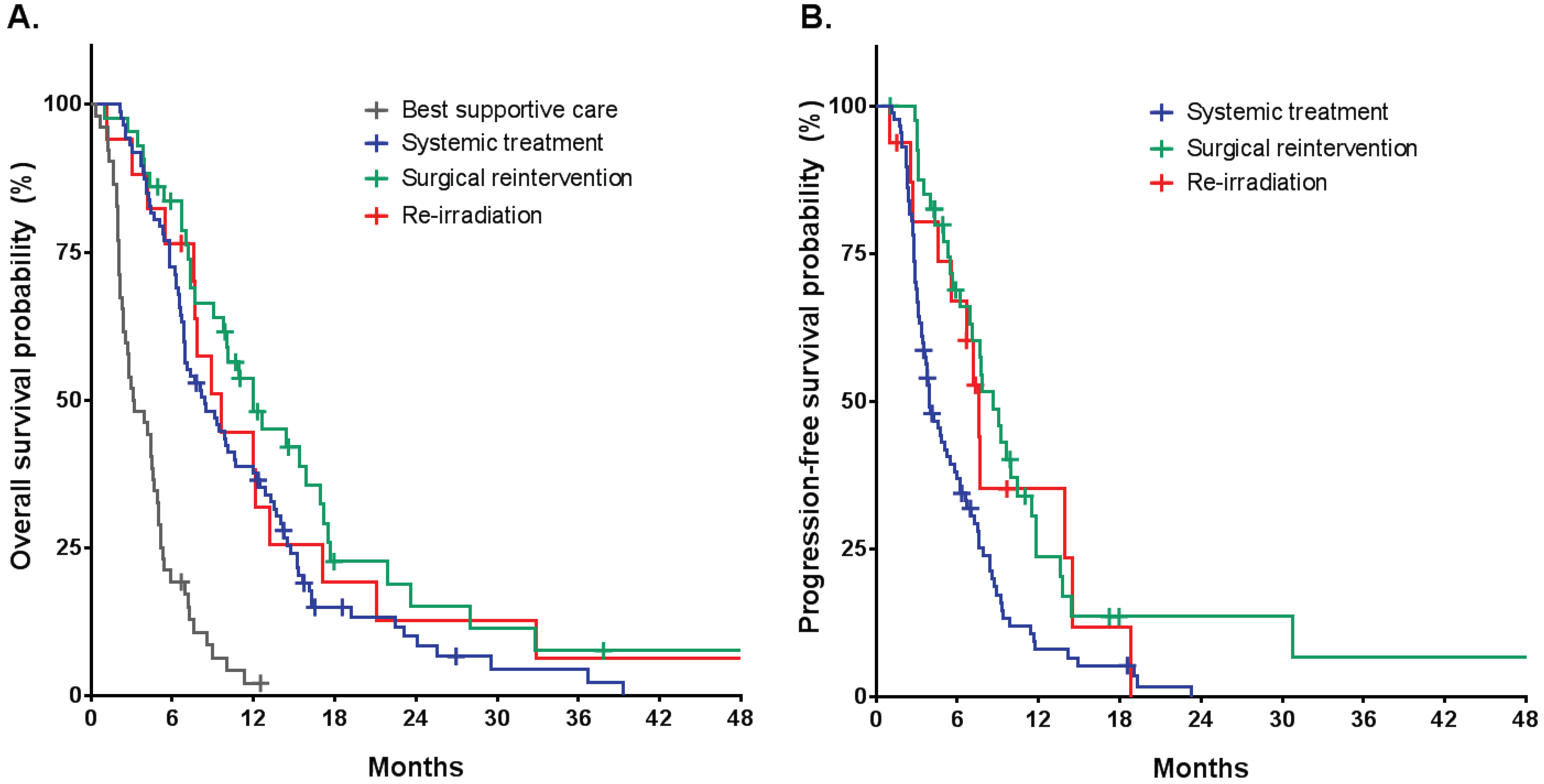

Supplement: Supplementary file 2 — Supplementary material 2 (PDF 160 KB) [file 11060_2017_2564_MOESM2_ESM.pdf]
