# Supplementary material for: Treatment outcome of patients with recurrent glioblastoma multiforme: a retrospective multicenter analysis
Source: J Neurooncol. 2017 Jul 20;135(1):183–92. doi: 10.1007/s11060-017-2564-z (PMC5658463; doi:10.1007/s11060-017-2564-z)
Supplement: Supplementary file 3 — Supplementary material 3 (DOCX 29 KB) [file 11060_2017_2564_MOESM3_ESM.docx]

**Treatment outcome of patients with recurrent glioblastoma multiforme: a retrospective multicenter analysis**

Myra E. van Linde^1,†^, Cyrillo G. Brahm^1,2,†^, Philip C. de Witt Hamer^3^, Jaap C. Reijneveld^4^, Anna M.E. Bruynzeel^5^, W. Peter Vandertop^3^, Peter M. van de Ven^6^, Michiel Wagemakers^7^, Hiske L. van der Weide^8^, Roelien H. Enting^9^, Annemiek M.E. Walenkamp^2^, Henk M.W. Verheul^1^

^1^Department of Medical Oncology, Cancer Center Amsterdam, VU University Medical Center, Amsterdam, the Netherlands

^2^ Department of Medical Oncology, University Medical Center Groningen, Groningen, the Netherlands

^3^ Department of Neurosurgery, Cancer Center Amsterdam, VU University Medical Center, Amsterdam, the Netherlands

^4^ Department of Neurology, Cancer Center Amsterdam, VU University Medical Center, Amsterdam, the Netherlands

^5^Department of Radiotherapy, Cancer Center Amsterdam, VU University Medical Center, Amsterdam, the Netherlands

^6^ Department of Epidemiology and Statistics, VU University Medical Center, Amsterdam, the Netherlands

^7^ Department of Neurosurgery, University Medical Center Groningen, Groningen, the Netherlands

^8^Department of Radiotherapy, University Medical Center Groningen, Groningen, the Netherlands

^9^ Department of Neurology, University Medical Center Groningen, Groningen, the Netherlands

^†^These authors contributed equally to this work.

**Corresponding author:**

Henk M.W. Verheul, MD, PhD, Professor of Medical Oncology, Chair Department of Medical Oncology, Cancer Center Amsterdam, VU University Medical Center, P.O. Box 7057, 1007 MB Amsterdam, the Netherlands, Phone: +31 20 444 4321, Fax: +31 20 444 4355, E-mail: [h.verheul@vumc.nl](mailto:h.verheul@vumc.nl)

| **Table S1. Cox proportional hazards model of progression-free survival after adjustments for confounders** | | | | |
| --- | --- | --- | --- | --- |
|  |  | **Study population (*n* = 175)** | | |
|  | **Factor** | **No. of Events / No. of Patients** | **HR (95% CI)** | ***P*  value** |
| **UNIVARIATE** | Treatment groups |  |  | < 0.001 |
|  | Systemic treatment | 95/104 | 1 |  |
|  | Surgical reintervention | 39/53 | 0.41 (0.27 - 0.62) |  |
|  | Re-irradiation | 14/18 | 0.64 (0.36 - 1.14) |  |
|  |  |  |  |  |
| **MULTIVARIATE ANALYSIS** | Treatment groups |  |  | < 0.001 |
|  | Systemic treatment | 95/104 | 1 |  |
|  | Surgical reintervention | 39/53 | 0.39 (0.23 - 0.59) |  |
|  | Re-irradiation | 14/18 | 0.76 (0.41 - 1.42) |  |
|  | Age (years) | - | 0.99 (0.98 - 1.01) | 0.302 |
|  | Sex |  |  | 0.001 |
|  | Male | 103/121 | 1 |  |
|  | Female | 45/54 | 0.53 (0.35 - 0.78) |  |
|  | Tumor extent |  |  | 0.288 |
|  | Single lobe | 112/131 | 1 |  |
|  | Multiple lobes | 36/44 | 1.26 (0.83 - 1.91) |  |
|  | Extent of initial resection |  |  | 0.662 |
|  | Incomplete | 88/104 | 1 |  |
|  | Complete | 43/52 | 0.83 (0.56 - 1.24) |  |
|  | Recurrence-free interval (days) | - | 1.00 (0.999 - 1.00) | 0.276 |
|  | ECOG performance score |  |  | 0.042 |
|  | ECOG 0 | 56/65 | 1 |  |
|  | ECOG 1 | 78/89 | 1.66 (1.11 - 2.47) |  |
|  | ECOG 2 | 12/18 | 0.91 (0.46 - 1.80) |  |
|  | ECOG 3 | 2/3 | 1.03 (0.23 - 4.63) |  |
|  | Use of steroids |  |  | 0.106 |
|  | No | 72/86 | 1 |  |
|  | Yes | 73/86 | 1.56 (1.03 - 2.36) |  |

| **Supplementary table S2. Survival outcomes of completed Stupp patients** | | | |
| --- | --- | --- | --- |
|  | **Study population (*n =* 199)** | | |
|  | **Proportion of patients - no (%)** | **Median OS (95% CI)** | **Median PFS (95% CI)** |
| Treatment groups |  |  |  |
| Best supportive care | 52 / 199 (26,1%) | 3.1 months [1.5 – 4.7 months] | - |
| Systemic treatment | 87 / 199 (43,7%) | 8.4 months [6.1 – 10.7 months] | 3.9 months [3.0 – 4.9 months] |
| Surgical reintervention | 43 / 199 (21,6%) | 12.0 months [7.4 – 16.6 months] | 8.7 months [6.9 – 10.5 months] |
| Re-irradiation | 17 / 199 (8,5%) | 9.7 months [6.2 – 13.1 months] | 7.6 months [6.1 – 9.2 months] |

| **Supplementary table S3. Multivariate Cox regression analysis of overall survival in completed Stupp patients** | | | |
| --- | --- | --- | --- |
|  | **Study population (*n* = 199)** | | |
| **Factor** | **No. of Events / No. of Patients** | **HR (95% CI)** | ***P* value** |
| Treatment groups |  |  | < 0.001 |
| Best supportive care | 50 / 52 | 1 |  |
| Systemic treatment | 80 / 87 | 0.28 (0.17 - 0.47) |  |
| Surgical reintervention | 33 / 43 | 0.24 (0.12 - 0.46) |  |
| Re-irradiation | 15 / 17 | 0.37 (0.18 - 0.74) |  |
| Age (years) | - | 1.00 (0.99 - 1.02) | 0.487 |
| Sex |  |  | 0.009 |
| Male | 126 / 135 | 1 |  |
| Female | 52 / 64 | 0.62 (0.43 - 0.89) |  |
| Tumor extent |  |  | 0.005 |
| Single lobe | 133 / 149 | 1 |  |
| Multiple lobes | 45 / 50 | 1.72 (1.18 - 2.50) |  |
| Extent of initial resection |  |  | 0.723 |
| Incomplete | 112 / 128 | 1 |  |
| Complete | 48 / 54 | 0.93 (0.65 - 1.33) |  |
| Recurrence-free interval (days) | - | 0.999 (0.999 - 1.00) | 0.012 |
| ECOG performance score |  |  | 0.010 |
| 0 | 49 / 60 | 1 |  |
| 1 | 76 / 86 | 1.45 (0.95 - 2.22) |  |
| 2 | 31 / 31 | 1.36 (0.77 - 2.42) |  |
| 3 | 22 / 22 | 3.37 (1.60 - 7.11) |  |
| Use of steroids |  |  | 0.001 |
| No | 71 / 88 | 1 |  |
| Yes | 101 / 103 | 1.85 (1.22 - 2.80) |  |
